# Characterization of stroke-related upper limb motor impairments across various upper limb activities by use of kinematic core set measures

**DOI:** 10.1186/s12984-021-00979-0

**Published:** 2022-01-12

**Authors:** Anne Schwarz, Miguel M. C. Bhagubai, Saskia H. G. Nies, Jeremia P. O. Held, Peter H. Veltink, Jaap H. Buurke, Andreas R. Luft

**Affiliations:** 1 Vascular Neurology and Neurorehabilitation, Department of Neurology, University Hospital Zurich, University of Zurich, Zurich, Switzerland; 2 Biomedical Signals and Systems (BSS), University of Twente, Enschede, The Netherlands; 3 Cereneo, Center for Neurology and Rehabilitation, Vitznau, Switzerland; 4 Roessingh Research and Development B.V., Enschede, The Netherlands

**Keywords:** Biomechanical phenomena, Kinematics, Upper extremity, Stroke

## Abstract

**Background:**

Upper limb kinematic assessments provide quantifiable information on qualitative movement behavior and limitations after stroke. A comprehensive characterization of spatiotemporal kinematics of stroke subjects during upper limb daily living activities is lacking. Herein, kinematic expressions were investigated with respect to different movement types and impairment levels for the entire task as well as for motion subphases.

**Method:**

Chronic stroke subjects with upper limb movement impairments and healthy subjects performed a set of daily living activities including gesture and grasp movements. Kinematic measures of trunk displacement, shoulder flexion/extension, shoulder abduction/adduction, elbow flexion/extension, forearm pronation/supination, wrist flexion/extension, movement time, hand peak velocity, number of velocity peaks (NVP), and spectral arc length (SPARC) were extracted for the whole movement as well as the subphases of reaching distally and proximally. The effects of the factors gesture versus grasp movements, and the impairment level on the kinematics of the whole task were tested. Similarities considering the metrics expressions and relations were investigated for the subphases of reaching proximally and distally between tasks and subgroups.

**Results:**

Data of 26 stroke and 5 healthy subjects were included. Gesture and grasp movements were differently expressed across subjects. Gestures were performed with larger shoulder motions besides higher peak velocity. Grasp movements were expressed by larger trunk, forearm, and wrist motions. Trunk displacement, movement time, and NVP increased and shoulder flexion/extension decreased significantly with increased impairment level. Across tasks, phases of reaching distally were comparable in terms of trunk displacement, shoulder motions and peak velocity, while reaching proximally showed comparable expressions in trunk motions. Consistent metric relations during reaching distally were found between shoulder flexion/extension, elbow flexion/extension, peak velocity, and between movement time, NVP, and SPARC. Reaching proximally revealed reproducible correlations between forearm pronation/supination and wrist flexion/extension, movement time and NVP.

**Conclusion:**

Spatiotemporal differences between gestures versus grasp movements and between different impairment levels were confirmed. The consistencies of metric expressions during movement subphases across tasks can be useful for linking kinematic assessment standards and daily living measures in future research and performing task and study comparisons.

*Trial registration*: ClinicalTrials.gov Identifier NCT03135093. Registered 26 April 2017, https://clinicaltrials.gov/ct2/show/NCT03135093.

## Introduction

The human upper limb can be expressed by seven main degrees of freedom, excluding the hand, that allow for highly variable movements and interactions with the environment. After stroke, this movement complexity can be affected due to a disruption in the cerebral sensorimotor networks that lead to inefficient or abnormal movement activation [1]. Sensitive assessments of the motor function on the level of movement quality and influences of deficits on daily life functionality are important to reveal relevant movement limitations and drive interventions for improving functional restoration [2].

Over the last decades, upper limb kinematic assessments have increasingly been used as primary or secondary outcome measures, next to standard clinical assessments in randomized-controlled trials concerning stroke rehabilitation [3–16]. Upper limb kinematic assessments have been investigated to test the effectiveness of different therapies, namely constraint induced movement therapy [14–16], trunk restraint training [17], robotic-assisted training [18–20], virtual reality training [21, 22], bilateral arm training [23–25], Botulinum toxin [26] and mirror therapy [27]. This tendency demonstrates the additional value of kinematic assessments to complement the standard clinical assessments and their broad evaluation level of movement quality. The advantage of upper limb kinematic measurements, compared to standard clinical assessments, is that different aspects of motion can be tracked objectively and continuously [28]. Alongside with this emerging field, the variability and heterogeneity of kinematic assessment protocols and chosen outcomes increased, making it difficult to interpret findings across studies [29]. A systematic review of upper limb kinematic assessments and metrics in subjects after stroke published criteria regarding upper limb assessment protocols. Out of 151 different metrics, task / movement time, path length ratio, number of velocity peaks, shoulder flexion/extension angle, trunk displacement, and peak velocity were proposed as core set metrics for facilitating the standardization and comparability of upper limb kinematic analysis after stroke [30]. These core set metrics performed best in terms of usage frequency, validity and/ or reliability. It has further been shown that upper limb kinematic measurements after stroke were frequently assessed in relatively fixed measurement surroundings such as camera-based motion laboratories or robot-based measurement systems. These measurements have the strong disadvantage that the movements the patients have to perform are device-specific and often restricted to simple reach-to-point, or tracking motions [30]. It is questionable, to what extent, a device-restraint planar pointing task is representative for movement tasks in daily living. It is unknown, if the movement characteristics of these different tasks are different for the same person as well as between people performing the same task or not. A significant impact of the movement task content and contexts on motor planning and behavior has been reported for pointing, or grasping of simulated or real objects [31, 32] and might be one of the biggest barriers in the interpretation and overall comparison of upper limb movement kinematics. With the exception of some natural tasks such as the standardized task of drinking from a glass [33], it is unknown how the outcomes of the current kinematic assessments relate to real-life performance and other similar functional upper limb movement tasks.

To overcome the described issue of highly variable and complex movements of the human upper limb, effort has been put into the development of a taxonomy for upper limb motion that subdivides motions based on the complexity and duration into activities, functional movements, and functional primitives [34]. The functional movement primitives or movement subphases, such as reaching or transporting, were suggested to be seen as building blocks or even more granular elements of motion, that are consistent across movements [35]. Based on this theorem, it could be assumed that for example reaching to grasp a glass is based on the same primitive or building block as reaching to grasp a phone receiver. Observing upper limb motions on the level of the functional movement primitives or movement subphases could thereby enable across task comparisons of movement quality and overcome issues of anatomical and task-related complexity. In person with stroke-related upper limb impairments, the kinematic analysis on the level of movement subphases could thus help to uncover pathophysiological mal-adaptations and relevant limitations in movement behavior such as diminished elbow extension during reach or arm elevation and hand speed during object transport.

Most of the pre-mentioned research on upper limb kinematic assessments was based on one or two movement tasks narrowing the findings down to a specific type of movement. A wider set of upper limb assessment activities, including non-contact movements such as gestures, or contact movements such as grasping activities, would increase the representativeness and comprehensiveness of the kinematic characterization of upper limb movement quality in daily life. In the present study, a set of 20 movement tasks representative for activities of daily life was used covering the main requirements of movement control of the human upper limb degrees of freedom (DOF) in terms of workspace, grasp configuration, interaction with the environment and complexity [36]. The aim of the study is to characterize and differentiate stroke-related upper limb function and impairment for movements related to activities of daily life. To this end, kinematics presenting the main upper limb spatiotemporal movement characteristics were recorded during gesture and grasping actions.

The first question is whether kinematic characteristics are different between movement types of no-contact-based gesture movements and contact-based grasping. The second question is whether significant effects can be found related to subgroups of no, mild and moderate upper limb impairment. The third question is attributed to the comparability of movement subphases. It is questioned whether phases of reaching distally towards ipsilateral maximum arm length and reaching or transporting proximally towards the head are consistent in terms of spatiotemporal kinematic expressions and relations across different movement tasks and impairment levels.

## Methods

A prospective cross-sectional observational study on subjects after chronic stroke and healthy subjects was performed to explore the relationship between upper limb function and activities as measured by clinical assessments and by a wearable sensor-based motion capture system. The study took place between July 2017 and October 2019 at the rehabilitation clinic cereneo (Vitznau, Switzerland).

### Study participants

The study sample consisted of 26 subjects with a unilateral ischemic or hemorrhagic stroke in the chronic stage (> 6 months) with presence of partial upper limb motor impairment, nonetheless with the ability to lift the arm against gravity (> 30° of shoulder flexion) and to flex and extend the fingers for basic grasp performances. The subjects were excluded, if increased upper limb muscle tone inducing limitations in range of motion (modified Ashworth Scale ≥ 3 in one of the tested muscle groups), severe sensory deficits in the upper limb (Erasmus modifications to the revised Nottingham Sensory Assessment of 0 in one of the test regions), or upper limb impairments unrelated to the neurological disease, such as preexisting orthopedic problems were present. Five age-matched healthy subjects without history of neurologic impairments nor limitations in upper limb movements were included for the acquisition of kinematic reference data. Each participant had to be able to understand and follow basic commands to perform the study experiments and to give written informed consent before inclusion, according to the Declaration of Helsinki and the Swiss regulatory authorities (BASEC-ID: 2016-02075).

### Study experiments

All experiments were performed by an experienced research therapist during a single-day measurement per subject at the rehabilitation clinic cereneo (Vitznau, Switzerland). After donning and calibration of the inertial sensor system, each participant was asked to perform a set of upper limb activities three times. Stroke participants were asked to perform the movements with the affected upper limb, healthy participants used their non-dominant side.

During the experiment study participants were sitting in upright position on an armless chair. The start and end position for the gesture movements were defined with the tested arm drooped straight with the hand approximately above the hip. The grasping movements were performed on a height-adjustable table with the start and end position of the tested arm defined by 90° of elbow flexion and neutral shoulder position. The corresponding position of the index fingers on the table was marked as a reference for each subject. After finishing the task, the participant was asked to return to the start position.

**Fig. 1:**
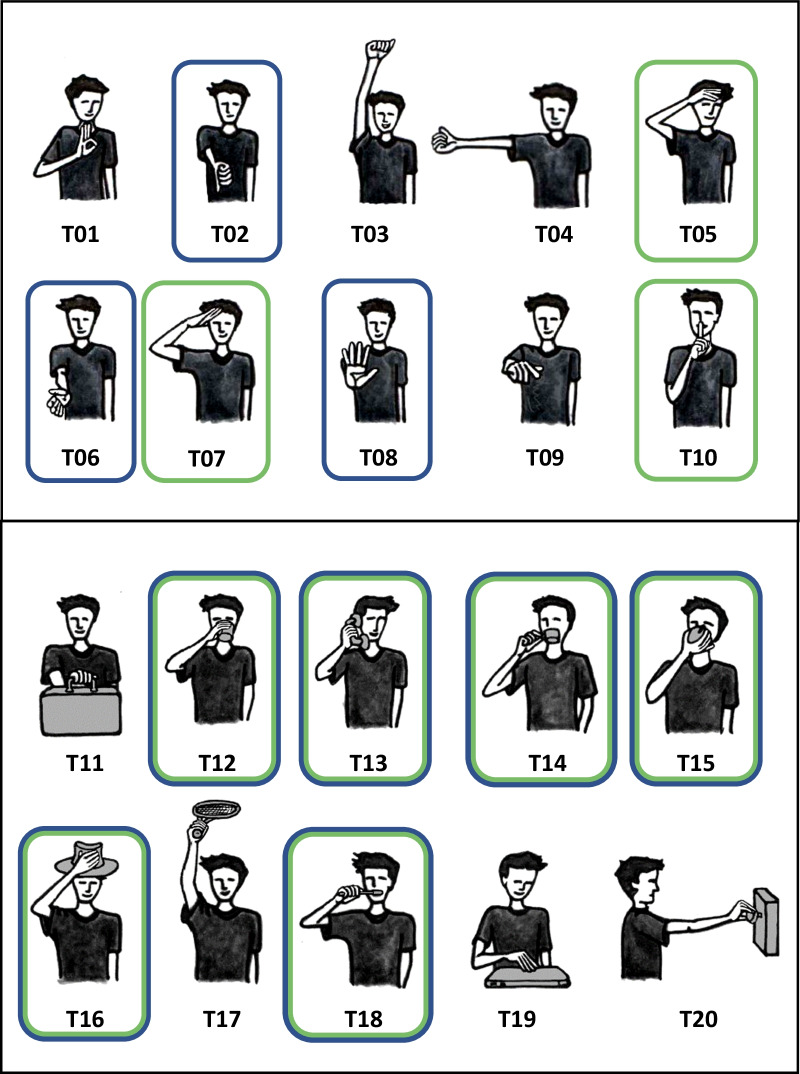
Experimental protocol of 20 activities of daily life. The task items T1–T10 include intransitive gesture movements without object contact. The task items T11–T20 represent transitive grasping movements with objects contact and manipulation. Tasks that were included in the subphase analysis of reaching distally are encircled in blue and those that include reaching proximally are encircled in green

The experimental task selection is based on previous works [36, 37], consisting of ten intransitive, gesture movements, and ten transitive, reach-to-grasp, and manipulation movements, as illustrated in Fig. 1 and described in detail in Table 1. This task set covers the main upper limb movement workspace and contains the main grasping types [38], while enabling the differentiation between gesture movements without contact and grasping actions with object contact and manipulation.

**Table 1:** Set of daily living tasks

#	Motion description	Contact	Subphase analysis
1	OK gesture	No	
2	Thumb down (lifting)	No	Reaching distally
3	Exultation (extending the arm up in the air and keeping it in with closed fist)	No	
4	Hitchhiking (extended elbow along the frontal plane, closed fist, thumb up)	No	
5	Block out sun from own face (with open hand, touch the face covering the eyes)	No	Reaching proximally
6	Greet (with open hand, moving wrist) (3 times)	No	Reaching distally
7	Military salute (with lifted elbow)	No	Reaching proximally
8	Stop gesture (extending the arm along the sagittal plane, parallel to the floor, with extended elbow, open palm)	No	Reaching distally
9	Pointing (with index finger) of something straight ahead (with outstretched arm)	No	
10	Silence gesture (bringing the index finger, with the remainder of the hand closed on the lips)	No	Reaching proximally
11	Reach and grasp a small suitcase (placed along own frontal plane) from the handle, lift it and place it on the floor (close to own chair, along own sagittal plane)	No	
12	Reach and grasp a glass, drink for 3 sec. and place it in the initial position	Yes 3^a^	Reaching distally transport proximally
13	Reach and grasp a phone receiver (placed along own sagittal plane), carry it to own ear for 3 sec. and place back	Yes 4^a^	Reaching distally transport proximally
14	Reach and grasp a small cup from the handle (2 fingers + thumb), drink for 3 sec. and place it in the initial position	Yes 8^a^	Reaching distally transport proximally
15	Reach and grasp an apple, mimic biting and put it in the initial position	Yes 11^a^	Reaching distally transport proximally
16	Reach and grasp a hat (placed on the right side of the table) from its top and place it on own head	Yes 12, 13^a^	Reaching distally transport proximally
17	Reach and grasp a tennis racket (placed along own frontal plane) and play a forehand (the subject is still seated)	Yes 2, 3, 4^a^	
18	Reach and grasp a toothbrush, brush teeth, and put the toothbrush inside a cylindrical holder	Yes 5^a^	Reaching distally transport proximally
19	Reach and grasp a laptop and open the laptop (without changing its position) (4 fingers + thumb)	Yes 6^a^	
20	Reach and grasp a doorknob (disk shape), turn it clockwise and counterclockwise	Yes 10^a^	

^a^Indicates the grasp type and number as classified by Cutcowski (1989)

From these 20 tasks, a subset of tasks with similar workspace demands was chosen to test similarities of motion primitives across tasks. We defined and tested four primitives, (1) reaching distally to grasp, (2) reaching distally to gesture, (3) reaching proximally to transport, and (4) reaching proximally to gesture. As described by Schambra et al. reaches are defined by motions with the intention to make contact with an object or target that might include contact in terms of grasp or touch at the end of movement [34]. The motion primitive of transport is defined by the purpose to convey an object that can result in motion away from the body or towards the body or head specifically [34]. In the present study, relevant workspace directions were further differentiated, such as reaching towards maximum armlength reaching distance and reaching towards the head, as indicated in the task representations in Fig. 1. Subphases of reaching towards an object placed in ipsilateral arm length distance and transporting towards the mouth, ear, or head can be detected in the tasks, drinking from a glass (T12, T14), taking the phone for a call (T13), biting into an apple (T15), putting on a hat (T16), and tooth brushing (T18). Therefore, recorded kinematics of reaching distally to grasp (1) and proximally to transport (3) were investigated across the tasks, T12, T13, T14, T15, T16, and T18. The associated tasks to reaching to gesture distally (2) are the thumb down (T02), greeting (T06), and the stop gesture (T08). Reaching proximally to gesture (4) is tested in the tasks, protecting the face from the sun (T05), the military salute (T07), and the silence gesture (T10).

### Measurement system

**Fig. 2:**
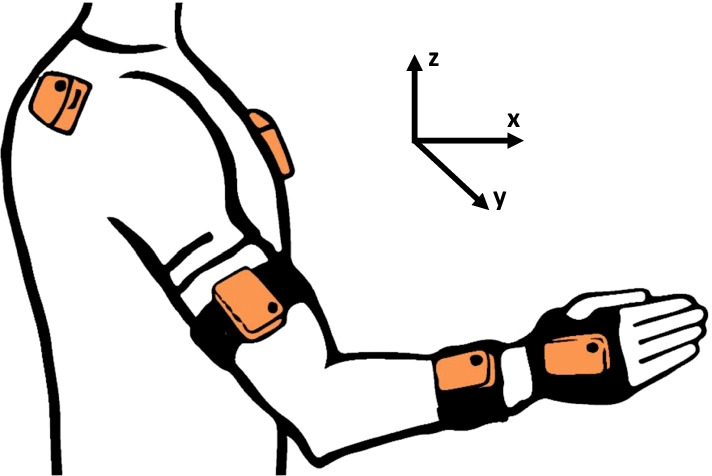
Upper limb sensor set-up. The location of the wearable sensors on predefined body segments is shown in relation to the global frame

For primary outcome measures, kinematic data was recorded by use of a full-body, wearable motion capture system, Xsens MVN Awinda (Xsens Technologies, Enschede, The Netherlands). The system offers real-time visualization, playback and editing of human motion capture data by a set of 17 wireless sensors that were attached symmetrically onto predefined body-parts of the participant. The sensors included in the upper limb motion analysis of this study were limited to the upper body, located above the sternum, the shoulder blade, the upper arm, the forearm and the back of the hand, as illustrated in Fig. 2.

Each sensor contains 3D accelerometry, 3D gyroscopes and 3D magnetometers to calculate subject-specific hand workspace and joint angles and positions [39]. The motion axes are pre-defined with respect to the global frame, where X is defined along the sagittal plane, Y along the horizontal plane and Z along the fontal plane in or against the direction of gravity as shown in Fig. 2. Positive values indicate outward, forward, or upward directions and negative values inward, backward, or downward directions. The upper limb kinematics captured with the Xsens MVN Awinda, included the maximum reach distance and movement range in vertical direction that have shown to correlate with the Fugl-Meyer Assessment of Upper Extremity [40]. The kinematic parameters were also applied in everyday surroundings for providing objective measurements of upper limb movements [41, 42].

### Kinematic core set

The kinematic core set is comprised of 10 parameters, namely trunk displacement, shoulder flexion/extension, shoulder abduction/adduction, elbow flexion/extension, forearm pronation/ supination, wrist flexion/extension, movement time, peak velocity, the number of velocity peaks (NVP), and spectral arc length (SPARC). These 10 parameters are evaluated for the complete movement tasks and the four motion primitives (1–4) of reach and transport in the subject-specific workspace separately. Matlab (MATLAB version 2018b, The Mathwork, Natick, MA, USA) was used for data processing.

The range of motion in the shoulder, elbow and hand is defined by the scalar measure from minimum to maximum joint angle. As suggested in the MVN Xsens manual, the ZXY Euler rotation sequence is used to present the joint angles, where flexion/extension is defined around the Z-axis, abduction/adduction is defined around the X-axis and internal/external rotation is defined around the Y-axis. However, an exception is made for shoulder abduction/adduction angle, which is recommended to be read out by the Euler rotation sequence XZY, to reduce estimation errors due to gimbal lock [43, 44]. Shoulder flexion/extension is defined as the angle along the sagittal plane, determined by rotation of the upper arm around the z-axis of the Euler sequence ZXY along the sagittal plane. Shoulder abduction/adduction is defined by rotation of the upper arm around the y-axis of the Euler sequence XZY along the frontal plane. Elbow flexion/extension is defined by rotation of the lower arm around the x-axis. Forearm pronation/supination is defined around the y-axis and wrist flexion/extension by around the around the x-axis. Trunk compensation is measured by changes in position and orientation of the sternum sensor [45]. The changes were calculated by the Euclidean distance minus an offset given by the mean of the first 10 data points of the x-, y- and z-direction.

Outcomes focusing on temporal aspects of motion included movement time, speed, and smoothness metrics. Movement time was defined as the time between movement onset and movement end, by applying a threshold of 2% of the peak velocity [33] measured with the hand-sensor. Peak velocity is determined by the maximum of linear hand velocity in m/s along the three directions with respect to the global reference frame, that were summed up by square root of the sum of the three directions. Similarly, NVP were summed up for three directions, reflecting changes between acceleration and deceleration phases and thereby the smoothness of the movement profile. A velocity peak is defined as the data point that is larger than its two neighboring samples in the linear hand velocity profile. The NVP is applied dimensionless without a per time unit, and valid, however could lack sensitivity and reliability in case of measurement noise [46]. SPARC has been suggested for its robustness against measurement noise. SPARC was defined to reflect the spectral energy induced by unsmooth, saccadic motions [46].

### Classification of relevant movement phases or motion primitives

**Fig. 3:**
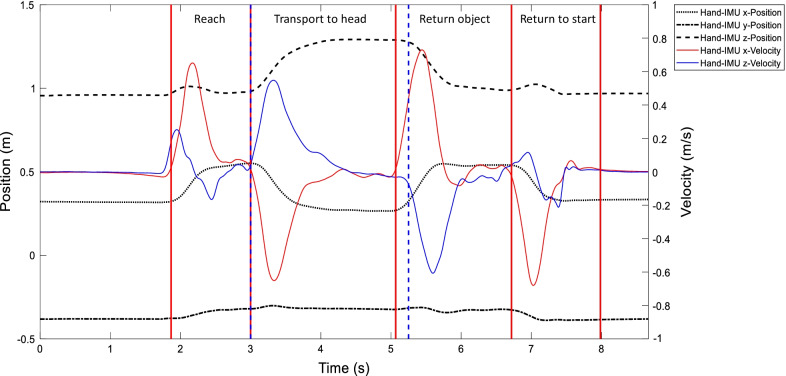
Feature-based movement phase segmentation for one subject and a single trial. One trial of the drinking task is presented by dimensionless plotting of the hand IMU position and velocity signals. The red lines indicate the threshold detection of velocity in x-direction. The blue dashed line refers to threshold detection of velocity in z-direction

As described in previous studies, segmentation of upper limb activities is important, to explore relevant aspects of task performance [33, 47, 48]. For the purpose of this study, the movement primitives, reach distally to grasp or gesture and reaching proximally to transport or gesture were further analyzed. Based on pre-assumption of a similar workspace across tasks, the phase segmentation was performed for the gesture movements T02, T05, T06, T07, T08, T10, and the grasp movements of the tasks T12, T13, T14, T15, T16, and T18 as highlighted in Fig. 1. Semi-automated phase detection was used by feature-based movement detection algorithms as exemplified in Fig. 3 for one trial of the drinking task (T12) of one subject. Similarly, feature-based segmentation has been used based on finger force detection and finger angular motions in reach-to-grasp movements [48], as well as based sensor signals of orientation angles in ambulation assessments [49].

The movement subphases of reaching distally to grasp or gesture towards maximum ipsilateral arm length were determined based on the maximum distance in position in x-direction along the sagittal plane and the velocity data of the hand-IMU. The subphases of reaching proximally to transport or gesture in direction towards the head were determined by the maximum position of the hand-IMU in z-direction along gravity vector, as well as the minimum position of the hand-IMU in x-direction. Reaching or transporting distally towards maximum ipsilateral armlength is detected from movement onset to maximum distance of the hand-IMU in x-direction, combined with a velocity threshold in x- and z-direction. Reaching or transporting proximally towards the head was defined by the hand-IMU trajectory maximum height in z-direction combined with a velocity threshold in the x- and z-direction. The automatic detection method focused on detecting the highest/lowest peaks in the velocity profile of the hand-IMU and identifying the beginning of increase in absolute velocity according to a threshold of 2% of the peak velocity in m/s [33]. The start of the ‘Reach’ corresponds to the first increase of the velocity of the hand IMU in the x-direction. The following point corresponds to the increase in the velocity of the sensor in the z direction, indicating the beginning of the movement towards the head (‘Transport to head’). The start of the ‘Return Object’ phase is identified by the increase of the velocity in the negative z-direction. Finally, the last phase (‘Return to start’) is identified via the last negative peak of the velocity profile of the sensor’s x-direction. For gesture movement, only three points were determined, the movement onset, target position, as defined by maximum hand distance x-direction and or maximum height in z-direction, and the movement end. The automatic phase-detection was verified by visual inspection one trial after another and corrected manually if needed.

### Statistical analysis

The statistical analysis was performed using Matlab (MATLAB version 2018b, The Mathwork, Natick, MA, USA) and SPSS (SPSS version 26.0, IBM Corp.,Armonk, N.Y., USA). All kinematic outcome parameters were tested for normal distribution by inspecting the histograms. Descriptive statistics of the kinematic outcome parameters were presented in mean and the 95% confidence intervals.

The first and second research question were addressed by performing a linear mixed effects model analysis for each kinematic outcome parameter to test for the impact of the factor gesture versus grasping movements and the factor of the impairment level as fixed effects. For the analysis of impairment-based expressions in kinematic outcomes, the participants were allocated to the no, mild or moderate impairment group based on the Fugl-Meyer Assessment of the Upper Extremity (FMMA-UE). The healthy subjects with a FMMA-UE full score of 66/66 points constituted the no impairment group. Subjects with a score ranging from 48 to 65 points were assigned to the mild impairment group and those with a score ranging from 32 to 47 points to the moderate impairment group [34, 50–53].

To address the third research question on comparability of kinematic expressions during movement subphases across different tasks and impairment subgroups, we considered the metrics during the subphases of reaching distally and proximally that were extracted from the tasks T02, T05, T06, T07, T08, T10, T12, T13, T14, T15, T16, and T18. Differences in metric expressions between tasks were tested with the Kruskal–Wallis test, a non-parametric version of the one-factor ANOVA for the subphases, reaching distally to grasp (1), reaching distally to gesture (2), reaching proximally to transport (3) and reaching proximally to gesture (4). Non-significant results in the Kruskal–Wallis test (p > 0.05) indicate that metric characteristics across tasks are similar. Additionally, correlations between the ten core set metrics of reaching distally and reaching proximally were explored visually in a correlation matrix for each task and group of impairment severity. Correlation coefficients of r ≥ 0.5 or r ≤ − 0.5 were defined as significant and compared across tasks and impairment severity.

## Results

Thirty-one subjects were included in the present analysis, with the participant characteristics presented in Table 2. Fourteen subjects were affected on their dominant hand. Three subjects missed one movement task item (S09 missed T03, S13 missed T09, and S23 missed T17) of the 20 prescribed actions. The whole dataset consisted of 775 kinematic sets of the affected upper limb, represented by the mean values of the three repetitions per subject and task that has been published online [54].

**Table 2:** Participant characteristics

Characteristic	No impairment (N = 5)	Mild impairment (N = 13)	Moderate imapirement (N = 13)
Gender, female/male	2/3	5/8	5/8
Mean age (SD), years	65.75 (10.72)	62.85 (13.43)	60.69 (11.58)
Mean body height (SD), cm	169.41 (7.47)	174.77(12.92)	172.85(8.97)
Mean BMI (SD), kg/m^2^	23.26 (2.18)	26.02 (4.46)	27.92 (3.92)
Paretic body side, left/right	–	7/6	5/8
Months since stroke^a^	–	13 (9–29)	24 (18–34)
Initial stroke severity NIHSS^a^	–	6 (6–10)	10 (6–15)
MoCA (0–30)^a^	–	27 (26–28)	26 (24–28)
MAS sum of the upper extremity (0–14)^a,b^	–	3 (2–4)	1 (0–1)
EmNSA-UE (0–40)^a^	–	38 (36–38)	39 (38–40)
FMMA-UE (0–66)^a^	–	40 (37–42)	55 (53–59)
FMMA-UE arm subsection (0–36)^a^	–	22 (21–24)	30 (29–33)
FMMA-UE wrist subsection (0–10)^a^	–	6 (5–6)	7 (6–8)
FMMA-UE hand subsection (0–14)^a^	–	9 (5–10)	14 (13–14)
FMMA-UE coordination subsection (0–6)^a^	–	4 (3–4)	5 (4–5)

*BMI* body mass index, *EmNSA* Erasmus modified version of the Nottingham Sensory Assessment, *FMMA-UE* Fugl-Meyer Motor Assessment of the Upper Extremity, *MAS* modified Ashworth Scale, *MoCA* Montreal Cognitive Assessment, *NIHSS* National Institutes of Health Stroke Scale, *L* left, *SD* standard deviation

^a^Indicates that values are presented in median (interquartile range)

^b^Indicates MAS scores between 1 and 2 for seven muscle groups

### Kinematic core set of the total task execution

As shown in Table 3, all kinematic parameters were statistically significantly different between gesture and grasp movements when considering the total task execution across subjects. Gestures resulted in larger shoulder joint motions and higher peak velocity when compared to the grasping movements. Grasping movements were associated with larger trunk motions, increased elbow and wrist flexion/extension ranges and forearm pronation/supination as well as an increased NVP.

**Table 3:** Linear mixed model results on kinematics for the factor movement task across subjects

	All movements	Gesture movements	Grasp movements	Significance
Trunk displacement in cm	5.2 (4.3–7.3)	2.9 (2.2–3.6)	7.1 (6.1–8.2)	p < 0.001*
Shoulder flex/ext in degrees	52.1 (45.0–60.2)	76.1 (70.3–81.8)	66.5 (62.4–70.6)	p < 0.001*
Shoulder abd/add in degrees	17.9 (15.7–24.0)	26.7 (24.3–29.3)	29.3 (27.5–31.0)	p = 0.035*
Elbow flex/ext in degrees	55.7 (46.3–63.8)	71.3 (65.3–77.4)	92.33 (89.0–95.6)	p < 0.001*
Forearm pro/sup in degrees	42.5 (31.5–54.8)	54.1 (49.1–59.1)	65.5 (58.2–66.9)	p = 0.008*
Wrist flex/ext in degrees	33.3 (23.6–41.7)	21.1 (19.2–23.0)	33.2 (30.0–36.3)	p < 0.001*
Movement time in seconds	6.7 (5.5–8.4)	3.43 (3.17–3.69)	7.05 (6.46–7.64)	p < 0.001*
Peak velocity in m/s	0.8 (0.8–1.0)	1.99 (1.81–2.18)	1.22 (1.13–1.31)	p < 0.001*
NVP	63.2 (49.6–77.2)	21.0 (17.8–24.2)	58.0 (49.4–66.5)	p < 0.001*
SPARC	− 4.2 (− 4.9 to (− 3.8))	− 3.3 (− 3.5 to (− 3.1))	− 3.6 (− 3.8 to (− 3.4))	p = 0.023*

*Abd/Add* abduction/adduction, *Flex/Ext* flexion/extension, *NVP* number of velocity peaks, *Pro/Sup* pronation/supination, *SPARC* spectral arc length

Kinematic parameters are represented by mean and (95% confidence interval) for the totally collected data and for the gesture and grasp activities separately

*Indicates statistically significant differences (p < 0.05) between gesture and grasp kinematics

**Fig. 4:**
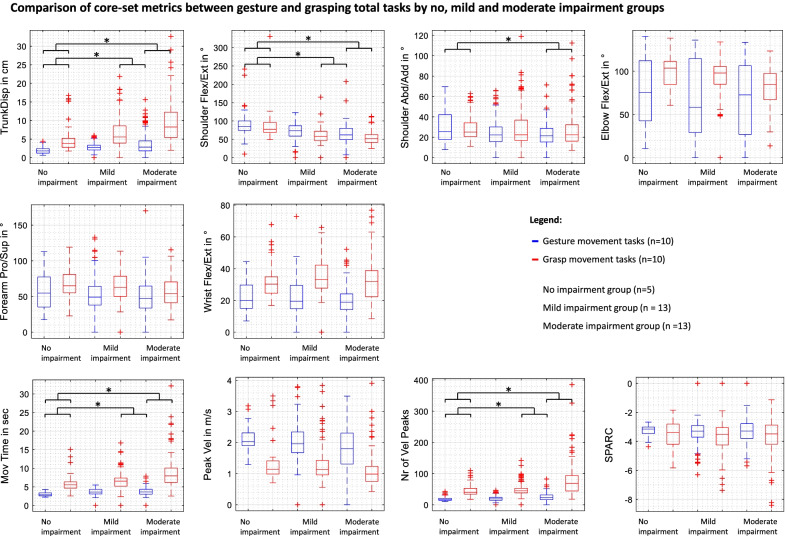
Effects of the task and impairment group on core set kinematics. *Abd/Add* abduction/adduction, *Flex/Ext* flexion/extension, *Pro/Sup* pronation/supination, *Mov* movement, *SPARC* spectral arc length, *TrunkDisp* trunk displacement, *Vel* velocity. *Indicates significant effects between the no, mild, and/or moderate impairment group for both gesture and grasp movements

On the level of subgroup comparisons between subjects with no, mild, and moderate impairments, each kinematic metric was included in a linear mixed model with the results indicated in Fig. 4. Significant effects of the impairment level were found for trunk displacement (p = 0.010) and shoulder flexion/extension (p = 0.001) with statistically significant post-hoc comparison between no impairment and moderate impairment, as well as between mild and moderate impairment for both kinematic outcomes. The interactions between the task and the impairment significantly influenced measures of shoulder abduction/adduction (p = 0.037). Movement time was significantly affected by the factor’s impairment level (p < 0.001), when the affected side is the dominant side (p = 0.038), and the interaction between the task and the impairment level (p < 0.001). Subjects of the no impairment group performed tasks faster with a mean of 4.2 (3.3–5.1) seconds, the mild impairment group with a mean of 5.2 (4.6–5.7) seconds and the moderate impairment group with a mean of 6.4 (5.9–7.0) seconds. In post-hoc analysis, significant differences were computed between the no and moderate impairment group (p < 0.001) as well as between the mild and moderate impairment group (p = 0.006). The NVP were shown to be influenced by the factor impairment level (p = 0.001) and the interaction between the task and the impairment group (p = 0.002). Significantly larger NVP were found in the moderate impairment group with a mean of 53.0 (45.5–60.4) when compared to the no impairment group (p = 0.012) with a mean of 31.1 (18.8–43.3) and between the moderate impairment group and the mild impairment group (p = 0.003) with a mean of 34.5 (27.0–41.9).

### Core set kinematics for subphases of reaching proximally and distally with and without contact

**Fig. 5:**
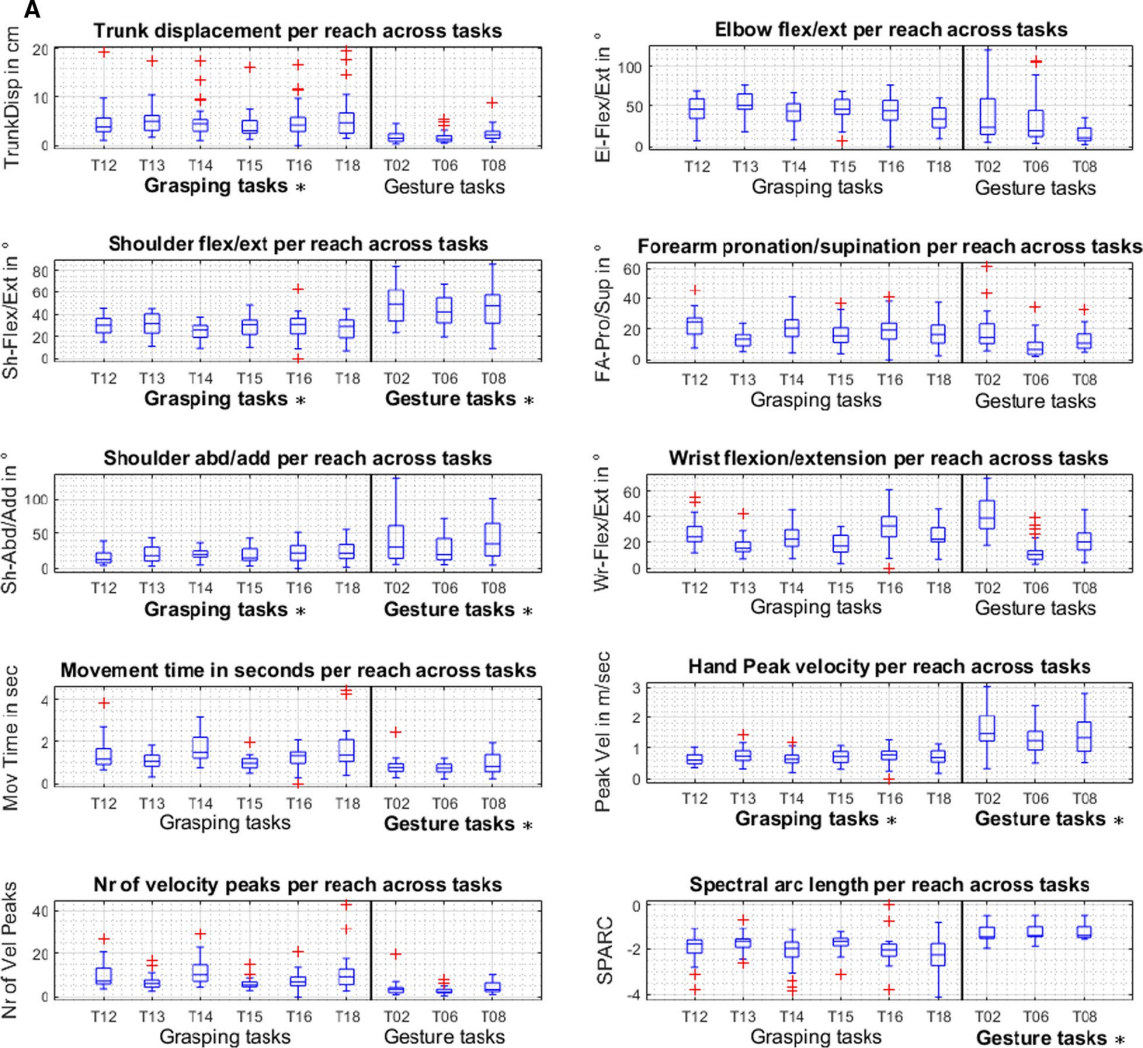

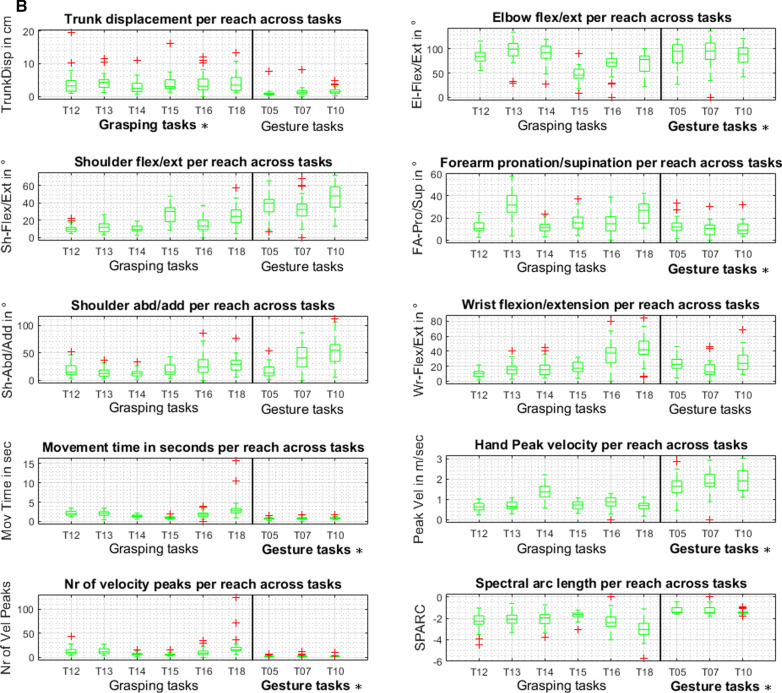
**A** Kinematic metrics during reaching distally per task across subjects (N = 31). The metric expressions are presented in boxplots per task. The vertical black line in each subplot separates the grasp movement tasks on the left side and gesture movement tasks on the right side. Statistically significant comparable metrics across tasks are indicated by asterisks and the bold notation of the gesture or grasping task caption. *El-Flex/Ext* elbow flexion/extension, *FA-Pro/Sup* forearm pronation/supination, *Mov Time* movement time, *No of Vel Peak* number of peak velocity, *Sh-Abd/Add* shoulder abduction/adduction, *Sh-Flex/Ext* shoulder flexion/extension, *SPARC* spectral arc length, *TrunkDisp* trunk displacement, *Wr-Flex/Ext* wrist flexion/extension. **B** Kinematic metrics during reaching proximally per task across subjects (N = 31). The metric expressions are presented in boxplots per task. The vertical black line in each subplot separates the grasp movement tasks on the left side and gesture movement tasks on the right side. Statistically significant comparable metrics across tasks are indicated by asterisks and the bold notation of the gesture or grasping task caption. *El-Flex/Ext* elbow flexion/extension, *FA-Pro/Sup* forearm pronation/supination, *Mov Time* movement time, *No of Vel Peak* number of peak velocity, *Sh-Abd/Add* shoulder abduction/adduction, *Sh-Flex/Ext* shoulder flexion/extension, *SPARC* spectral arc length, *TrunkDisp* trunk displacement, *Wr-Flex/Ext* wrist flexion/extension

Four classes of sub phases, reaching distally to grasp (1), reaching distally to gesture (2), reaching proximally to transport (3), and reaching proximally to gesture (4), were predefined and detected in the tasks T02, T05, T06, T07, T08, T10, T12, T13, T14, T15, T16, and T18. The automatic movement phase segmentation was verified without manual correction in 85% of the trials. Figure 5A illustrates the kinematic metric expressions while reaching distally towards the maximum reaching arm length (1, 2). The data is illustrated in one boxplot per task across subjects. Figure 5B represents the metrics during reaching proximally towards the person’s head (3, 4).

Statistical testing (Kruskal–Wallis test) of differences in metric expressions across different tasks of a movement subphase type resulted in statistically significant differences in metric expressions for the majority of conditions. As indicated with asterisks and the bold format of the gesture and grasping task subheadings in Fig. 5A, B, comparable metric expressions were found for trunk displacement in reaching to grasp distally (p = 0.235) and reaching to transport proximally (p = 0.413). Shoulder flexion/extension (p = 0.132) and shoulder abduction/adduction (p = 0.093) were found to be comparable in reaching to grasp distally and reach to gesture distally (shoulder flexion/extension, p = 0.613; shoulder abduction/adduction, p = 0.104). Elbow flexion/extension (p = 0.363) and forearm pronation/supination (p = 0.113) showed similar expressions in proximal reach gesture. Wrist flexion/extension was significantly different across tasks in all four movement subphase conditions. The temporal kinematics, movement time, peak velocity, and SPARC, were comparable between gestures distally as well as proximally with p-values ranging between p = 0.0866 and p = 0.290. Additionally, consistent results across tasks were found for peak velocity in reaching to grasp distally (p = 0.108) and for the NVP during reaching to gesture proximally (p = 0.127).

**Fig. 6:**
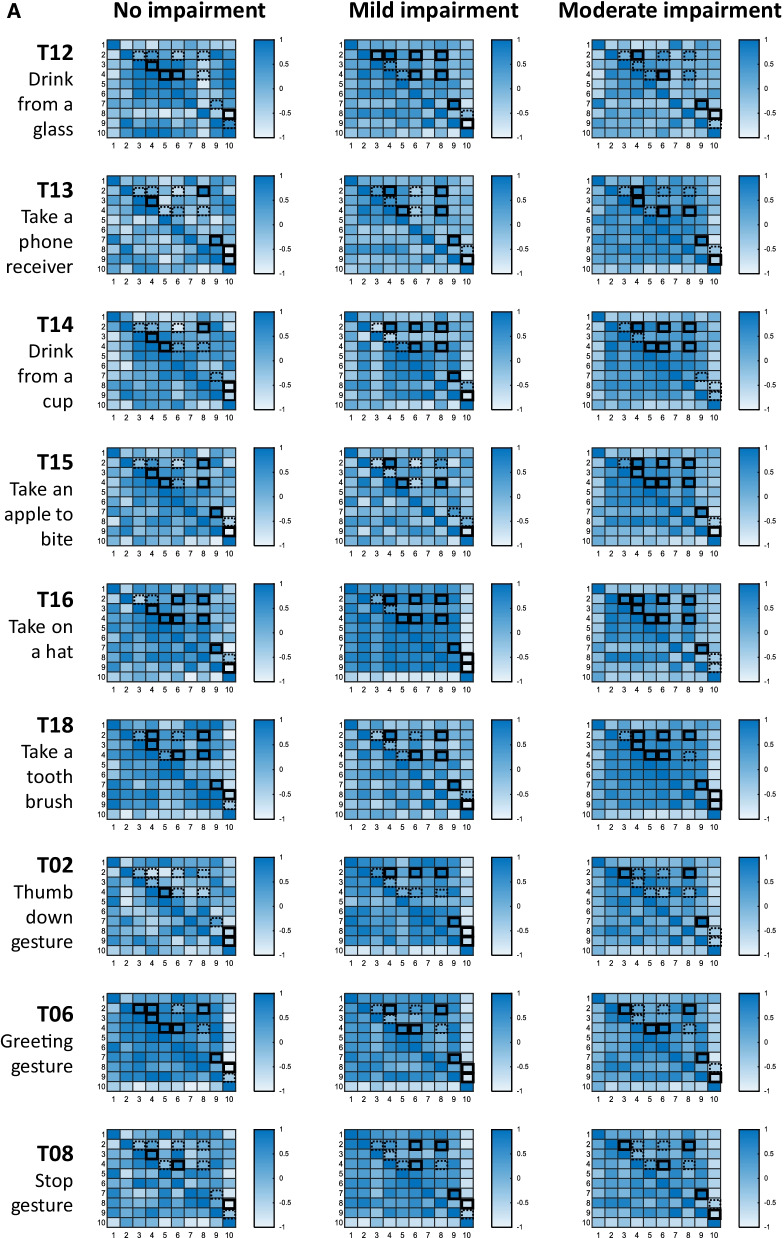

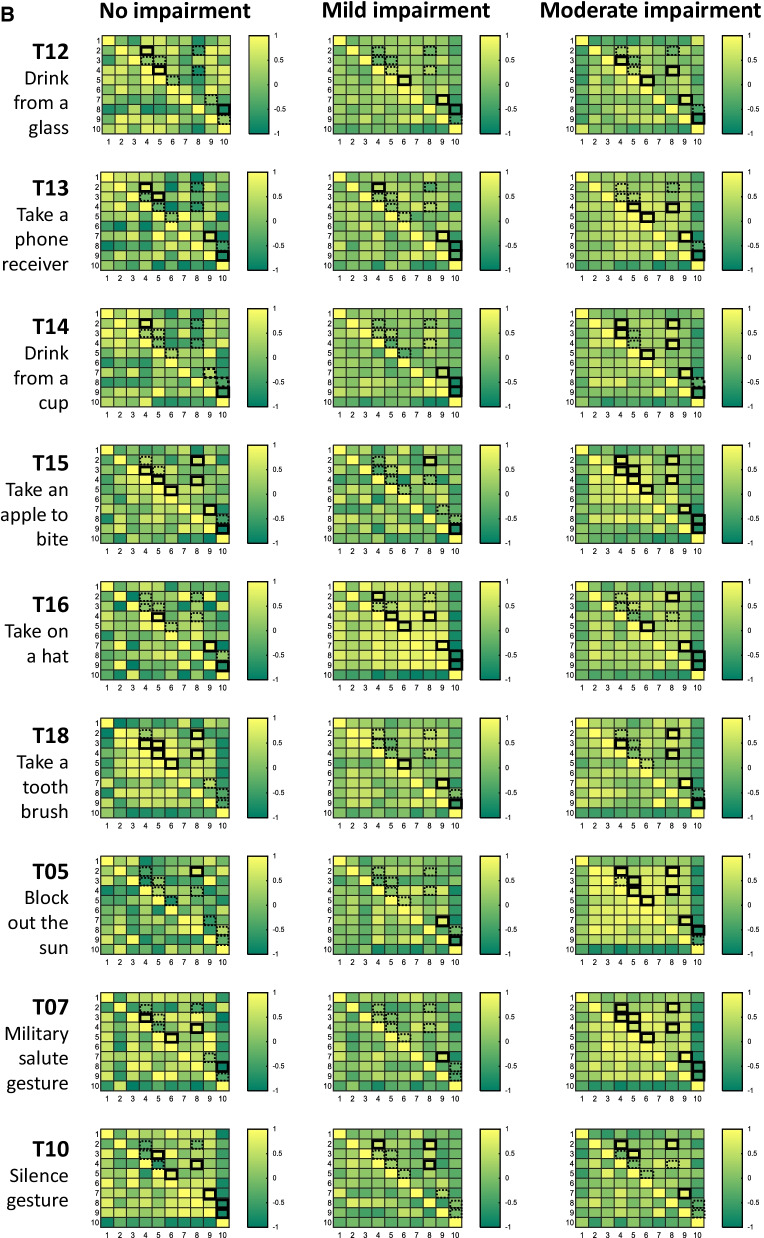
**A** Relationship between kinematics of reaching distally across subjects. Core set kinematics of reaching distally are correlated with each other per movement task and impairment level subgroups. 1, Trunk displacement; 2, Shoulder flexion/extension; 3, Shoulder abduction/adduction; 4, Elbow flexion/extension; 5, Forearm pronation/supination; 6, wrist flexion/extension; 7, Movement time; 8, Peak velocity; 9, Number of velocity peaks (NVP); 10, Spectral arc length (SPARC). The correlation coefficient is presented in a color code as shown on the right of each heat map. Strong correlations between metrics that were consistent across tasks are highlighted by black square outlines in the heatmap. The dashed black square outlines represent metric associations, suspected to be consistent across tasks, that were not significantly strong correlated. **B** Relationship between kinematics of reaching distally across subjects. Core set kinematics of reaching proximally are correlated with each other per movement task and impairment level subgroups. 1, Trunk displacement; 2, Shoulder flexion/extension; 3, Shoulder abduction/adduction; 4, Elbow flexion/extension; 5, Forearm pronation/supination; 6, wrist flexion/extension; 7, Movement time; 8, Peak Velocity; 9, Number of velocity peaks (NVP); 10, Spectral arc length (SPARC). The correlation coefficient is presented in a color code as shown on the right of each heat map. Strong correlations between metrics that were consistent across tasks are highlighted by black square outlines in the heatmap. The dashed black square outlines represent metric associations, suspected to be consistent across tasks, that were not significantly strong correlated

The relationship between the ten core set kinematic parameters during subphases of reaching distally and proximally were investigated in a correlation matrix for each task, and impairment subgroup. Figure 6 illustrates the strength of correlations for the no, mild and moderate impairment group in a heatmap for reaching distally in blue color code (Fig. 6A) and reaching proximally in green color code (Fig. 6B). Correlation coefficients of r ≥ 0.5, or r ≤ − 0.5 were set as cut-offs to determine significant associations between metrics. Significant associations are highlighted with black square outlines. Metric associations that were expected to be consistent across tasks but that failed to be significantly strong correlated are highlighted with dashed black square outlines.

During reaching distally, differences in terms of shoulder-elbow associations between the no impairment group and the mild and the moderate impairment group were found. Strong associations between elbow flexion/extension and shoulder abduction/adduction are observed across tasks in the no impairment group, whereas predominantly strong associations between elbow and shoulder flexion/extension are visible in the mild and the moderate impairment group. Across impairment groups, the following metric pairs were found to be strongly associated in most tasks; wrist flexion/extension and shoulder flexion/extension, peak velocity and elbow flexion/extension, peak velocity and shoulder flexion/extension, number of velocity peaks and movement time, and number of velocity peaks and spectral arc length.

During reaching proximally, stable relations across tasks were found between peak velocity and shoulder flexion/extension, between the number of velocity peaks and elbow flexion/extension, between the number of velocity peaks and movement time as well as between the NVP and the SPARC. A slight trend towards more consistent correlations is observed across tasks in the movement subgroups of reaching distally and proximally in the moderate impairment group.

## Discussion

This study aims to characterize upper limb movement behavior in subjects with and without stroke-related upper limb impairments when performing different activities including gestures and object-use that are associated with everyday life. Based on the presented results, task- and impairment specific kinematic expressions can be confirmed for the whole movement task execution and subphases of reaching distally and proximally that are summarized in the key findings of Box 1.

**Box 1:** Key findings on upper limb kinematic characteristics

Comparison of gesture and grasping movements •All spatiotemporal parameters differed significantly •Gestures are characterized by larger and faster motions in the shoulder joint •Grasp movements included larger trunk motions and joint ranges in forearm Pro/Sup and wrist Flex/Ext
Comparison of subjects with no, mild and moderate stroke-related impairments •Trunk displacement increased, shoulder Flex/Ext and Abd/Add decreased, movement time and the NVP increased with impairment •Interactions between task and impairment found for shoulder Abd/Add, movement time, NVP
Comparison and relations during reaching distally and proximally •Reaching distally was comparable in shoulder Flex/Ext, shoulder Abd/Add, movement time, hand peak velocity, the NVP and SPARC •Reaching proximally was comparable in trunk displacement, elbow Flex/Ext, forearm Pro/Sup, movement time, hand peak velocity, NVP and SPARC •Strong relations found across tasks between shoulder Flex/Ext, elbow Flex/Ext, hand peak velocity and between NVP and SPARC during reaching distally and proximally •Trends towards more strong and consistent associations between metrics with impairment might be attributable to less task-specific and selective motor abilities in subjects after stroke

*Abd/Add* abduction/adduction, *Flex/Ext* flexion/extension, *NVP* number of velocity peaks, *Pro/Sup* pronation/supination, *SPARC* spectral arc length

These study findings underpin the importance to consider task specificities in qualitative upper limb movement analysis, as already suggested in the pioneering works of Marc Jeannerod 50 years ago who found that the acceleration phase during reach-to-grasp is shorter compared to the deceleration phase in approach of the grasp, whereas during pointing movements the acceleration phase is considerably longer than the deceleration phase [55]. Furthermore, research suggests that movement tasks, such as rhythmic or discrete tasks, are controlled by different mechanisms [56, 57]. The presented findings on spatiotemporal movement characteristics of moderate to mildly affected stroke subjects in terms of increased trunk compensation and decreased peak velocity are in line with other research on movement kinematics of ADL movements, such as the drinking task assessment [51]. Effects of the task content and difficulty on kinematic outcome measures were detected across impairment severities, in the less-affected upper limb, as well as in healthy subjects [58, 59]. These findings support the consideration of the task difficulty in kinematic measurements is important to minimize floor and ceiling effects of the assessments. Thus, including similar gesture and grasping movements amongst other conditions in standardized assessment protocols would provide further insights into upper limb movement behavior.

Ten kinematic parameters, acquired and processed with a wearable sensing suit, were shown to be useful for assessing the spatiotemporal aspects of upper limb movement behavior for the total task performance as well as for the tasks subphases. The NVP showed good discriminability between different levels of upper limb impairment severity and different tasks. However, it has been criticized for its high task-dependency, in comparison to other smoothness measures, such as SPARC [46]. The strong and consistent relation between the number of velocity peaks and spectral arc length across movement subphases found in the present study, supports the assumption that both measures reflect the movement construct of smoothness. Other studies investigating smoothness measures suggested that normalized jerks are used as a unitless measure that normalizes for both amplitude and duration of the movement [60]. Log dimensionless jerk has been recommended for daily living recordings of different trial durations [61]. Although not explicitly addressed in the present study, the findings support the need to further explore the associations between different smoothness measures in relation to different populations and movement types that are representative for daily living.

Another topic, addressed herein, was the comparability of the metric expression across different tasks on the level of movement subphases. Even though preliminary, some trends and consistencies in movement characteristics of reaching distally and reaching proximally are described across different task types and impairment subgroups. These findings support the assumption of stereotypical motion primitives or building blocks as basic elements of functional motor tasks [35] and could potentially facilitate across task and study comparisons of upper limb kinematic measurements. Schambra et al. found that the two primitives or movement subphases of transport and reach were differentiable based on an unbiased machine learning algorithm with an accuracy of 92.1%. The algorithm’s nodes of the binary tree indicated greater wrist extension, wrist supination and elbow extension in reach, compared to less wrist extension and supination and more shoulder flexion and abduction during transport [34], which is comparable to the results of context-related differences in kinematic expressions in the present study. The algorithm was applied for phase segmentation was based on changes in the hand-sensor position and velocity in x- and z-direction. Visual inspection post-verification determined that the algorithm correctly carried out the segmentation in 85% of the included trials. Failures in automatic phase detection occurred frequently in datasets of subjects with more pronounced deficits that had irregular and slow movement profiles. We therefore conclude that the algorithm reliably detects movement sub phases of reaching to maximum arm length and the head or mouth. The reliable and time-saving segmentation and detection of movement sub phases in this controlled task set is an important prerequisite for the development of future real-life recording and analysis of upper limb functioning after stroke. Automatized movement phase segmentation and analysis has the potential to be useful for real-time feedback during training as well as for developments in assistive devices and training technology. The development of a hand orthosis is an example of the application of task-specific detection of upper limb movement kinematics. In this case, reaching movements with and without the intention to grasp by means of hand and finger sensors to develop the algorithm for controlling the hand orthosis [62]. Other examples of the application are found for specific training of the shoulder muscles, especially the rotator cuff [63] or assistive devices for arm weight support [4, 9, 10]. With respect to the stroke population, the development of devices improving the functionality and quality of upper limb movements after stroke is of great interest.

Some limitations need to be considered when interpreting the results. The number of healthy control subjects was small but well aligned with the stroke sample in terms of age distribution. The fact that this dataset [54] is part of a larger kinematic and kinetic datasets of similar experimental protocols [64] may help to gain a normative dataset on upper limb kinematics. We have not reported about the factor of hand dominance in the single task and subphase analysis. However, the factor of the affected is the dominant-side has been included in the linear-mixed model on gestures vs. grasping movement, without revealing significant effects on any of the tested kinematic parameters. Another limitation could be attributed to the lack of validation reference system for the sensor-based acquired kinematic measures. A systematic review on sensor-based joint angle estimations found measurement errors between 0.71° and 12.1° in the shoulder joint in comparison to optical systems with measurement errors around 1° [65]. Even so, sensor-based measurements were selected for recording the experimental protocol including object interactions and wide workspace contributions to circumvent problems of marker occlusion in optical systems and pave towards applications in flexible environments. Nevertheless, additional measures for movement detection, such as such as video recordings or sensors for interaction force detection, would be recommendable in future research to increase the accuracy of the automatic phase detection algorithm, thereby reducing the time and effort of manual data postprocessing.

## Conclusion

In conclusion, the analysis of gesture and grasp movements in a set of activities related to daily living revealed task-specific and impairment-specific characteristics in terms of different kinematic expressions. Grasping motions were characterized by more distally pronounced and slower motions that were less smooth and executed with larger trunk motions in stroke subjects when compared to faster gesture movement, that were shown to be less discriminative between impairment levels. We demonstrated that kinematic assessments of activities of daily living provide general and granular information on movement quality of relevant and natural upper limb motor activities. The comparison of subphases of reaching types across tasks revealed similarities in trunk compensation, shoulder motions and speed as well as smoothness-related metrics. These subphases additionally proved to be differently expressed between persons with no, mild and moderate stroke-related upper limb impairments.

## Data Availability

The datasets generated and used for the analysis presented in the current study are available in the Zenodo repository at: https://zenodo.org/record/3713449 [53].

## Abbreviations

Abd/add: Abduction/adduction
BMI: Body mass index
EmNSA: Erasmus modified version of the Nottingham Sensory Assessment
Flex/ext: Flexion/extension
FMMA-UE: Fugl-Meyer Motor Assessment of the Upper Extremity
MAS: Modified Ashworth Scale
MoCA: Montreal Cognitive Assessment
NIHSS: National Institutes of Health Stroke Scale
NVP: Number of velocity peaks
Pro/sup: Pronation/supination
SPARC: Spectral arc length
