# Correction to: Characterization of stroke-related upper limb motor impairments across various upper limb activities by use of kinematic core set measures

**DOI:** 10.1186/s12984-022-01048-w

**Published:** 2022-07-12

**Authors:** Anne Schwarz, Miguel M. C. Bhagubai, Saskia H. G. Nies, Jeremia P. O. Held, Peter H. Veltink, Jaap H. Buurke, Andreas R. Luft

**Affiliations:** 1 Vascular Neurology and Neurorehabilitation, Department of Neurology, University Hospital Zurich, University of Zurich, Zurich, Switzerland; 2 Biomedical Signals and Systems (BSS), University of Twente, Enschede, The Netherlands; 3 Cereneo, Center for Neurology and Rehabilitation, Vitznau, Switzerland; 4 Roessingh Research and Development B.V., Enschede, The Netherlands

## Correction to: Journal of NeuroEngineering and Rehabilitation (2022) 19:2 10.1186/s12984-021-00979-0

In Table 2 of this article [1], the 2nd and 3rd column name were mistakenly interchanged; this should appear as shown in this Correction (Table 2).

**Table 2:** Participant characteristics

Characteristic	Mild impairment (N = 5)	No impairment (N = 13)	Moderate imapirement (N = 13)
Gender, female/male	2/3	5/8	5/8
Mean age (SD), years	65.75 (10.72)	60.69 (11.58)	62.85 (13.43)
Mean body height (SD), cm	169.41 (7.47)	172.85 (8.97)	174.77 (12.92)
Mean BMI (SD), kg/m^2^	23.26 (2.18)	27.92 (3.92)	26.02 (4.46)
Paretic body side, left/right	–	5/8	7/6
Months since stroke*	–	24 (18–34)	13 (9–29)
Initial stroke severity NIHSS*	–	10 (6–15)	6 (6–10)
MoCA (0–30)*	–	26 (24–28)	27 (26–28)
MAS sum of the upper extremity (0–14)*^†^	–	1 (0–1)	3 (2–4)
EmNSA-UE (0–40)*	–	39 (38–40)	38 (36–38)
FMMA-UE (0–66)*	–	55 (53–59)	40 (37–42)
FMMA-UE arm subsection (0–36)*	–	30 (29–33)	22 (21–24)
FMMA-UE wrist subsection (0–10)*	–	7 (6–8)	6 (5–6)
FMMA-UE hand subsection (0–14)*	–	14 (13–14)	9 (5–10)
FMMA-UE coordination subsection (0–6)*	–	5 (4–5)	4 (3–4)

*BMI* Body Mass Index, *EmNSA* Erasmus modified version of the Nottingham Sensory Assessment, *FMMA-UE* Fugl-Meyer Motor Assessment of the Upper Extremity, *MAS* modified Ashworth Scale, *MoCA* Montreal Cognitive Assessment, *NIHSS* National Institutes of Health Stroke Scale, *L* left, *SD* standard deviation. *Values are presented in median (interquartile range); ^†^MAS scores between 1 and 2 for seven muscle groups

Also, the significance for Table 4 were mistakenly interchanged, figure 4 should appear as shown in this Correction (Fig. 4).

**Fig. 4:**
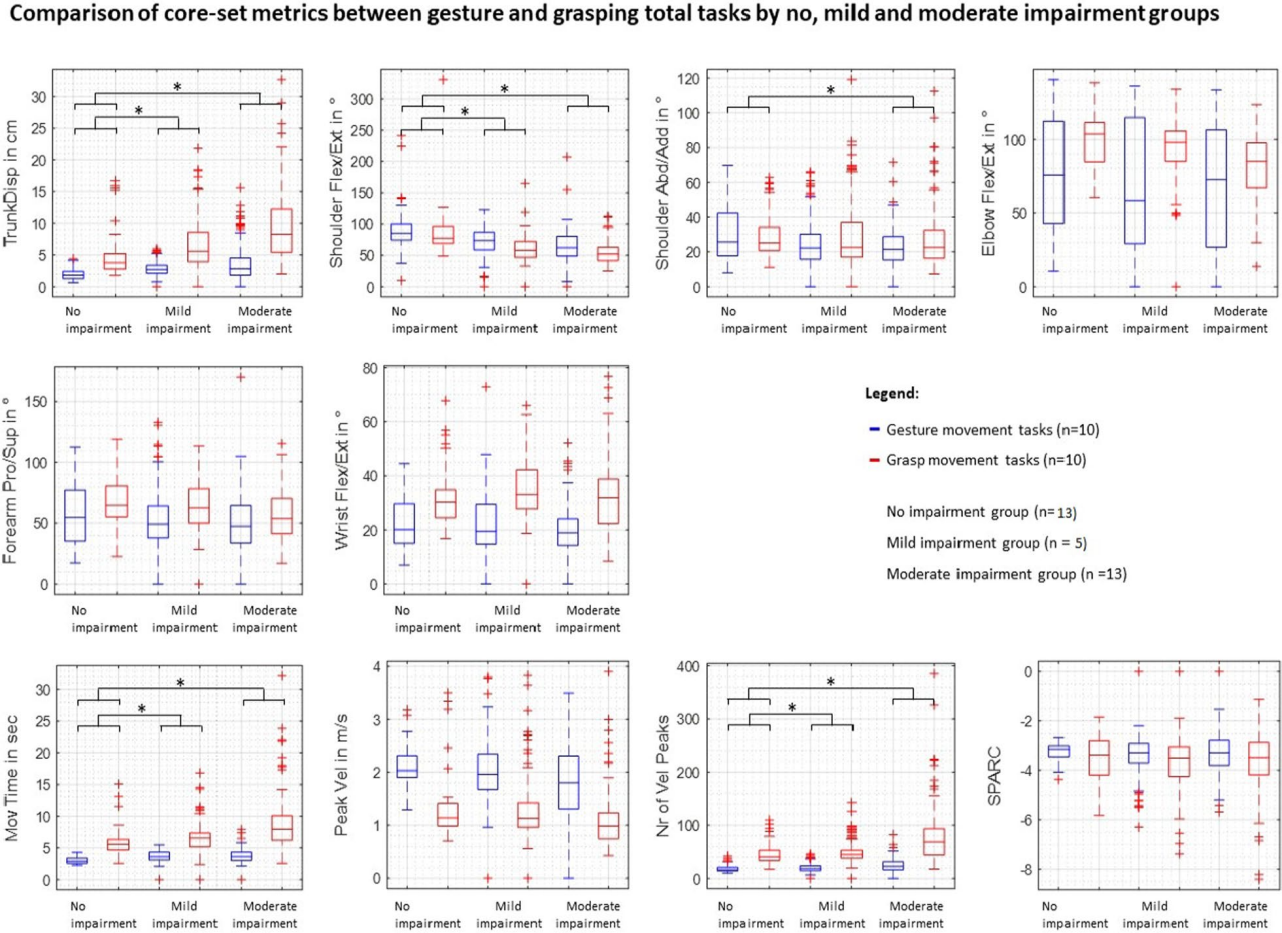
Efects of the task and impairment group on core set kinematics. *Abd/Add* abduction/adduction, *Flex/Ext* fexion/extension, *Pro/Sup* pronation/supination, *Mov* movement, *SPARC* spectral arc length, *TrunkDisp* trunk displacement, *Vel* velocity. * indicates signifcant efects between the no, mild, and/or moderate impairment group for both gesture and grasp movements

The original article has been corrected.
